# Do Medical Students Who Participate in a Research Gap Year Produce More Research During Residency?

**DOI:** 10.5435/JAAOSGlobal-D-21-00061

**Published:** 2021-05-13

**Authors:** Joshua Wright-Chisem, Matthew R. Cohn, JaeWon Yang, Daniel Osei, Monica Kogan

**Affiliations:** From the Hospital for Special Surgery, Orthopaedic Surgery, New York, NY (Dr. Wright-Chisem, and Dr. Osei), and the Department of Orthopaedic Surgery, Rush University Medical Center, Chicago, IL (Dr. Cohn, Yang, and Dr. Kogan).

## Abstract

The research gap year has become increasingly popular among medical students. It is also a well-known factor in consideration for orthopaedic surgery residency programs. Although medical students who participated in a research gap year typically enter residency with more research experience than their counterparts, it is unknown whether this translates to increased research productivity during residency compared with their peers. The purpose of this study was to investigate (1) whether residents who participated in a research gap year during medical school publish more peer-reviewed publications than their peers during residency, (2) whether residents who participated in a research gap year during medical school publish more first-author publications than their peers during residency, and (3) which applicant characteristics are associated with a greater number of peer-reviewed publications produced during residency.

**Methods:**

The number of peer-reviewed journal publications before and during residency was determined by querying PubMed for 81 orthopaedic surgery residents at two academic institutions. Electronic residency application service applications and curriculum vitae were reviewed to evaluate the number of conference podiums and conference posters presented before residency and during residency. The research productivity of residents who participated in a research gap year during medical school was compared with that of residents who had not participated in a research gap year. Multivariate regression was done to determine predictors of publishing peer-reviewed journal publications during residency.

**Results:**

Residents who participated in a research gap year during medical school produced more peer-reviewed journal publications during residency than those who did not (22.0 ± 20 versus 16.5 ± 20, *P* = 0.025). However, residents who participated in a research gap year did not produce more first-author publications compared with their peers (7.6 ± 10.0 versus 7.9 ± 7.0, *P* = 0.12). Residents who produced more publications before residency produced more publications while in residency (R = 0.363, *P* < 0.001). The United States Medical Licensing Examination step 1 score, medical school ranking, and sex were not associated with any difference in the number of journal publications produced during residency.

**Conclusion:**

A dedicated research year during medical school is associated with an increase in the number of peer-reviewed publications produced during residency. However, students who completed a research year did not publish more first-author publications than their peers. The number of publications before residency was a strong predictor of research output as a resident.

The process of selecting orthopaedic surgery residents is an ever-evolving task. It involves making an assessment of each applicant's traits—both subjective and objective—including United States Medical Licensing Examination (USMLE) score(s), medical school grades and reputation, away rotation performance, interview performance, and letters of recommendation, among many other factors.^1^ Because selection for an orthopaedic surgery residency becomes increasingly competitive and USMLE step 1 is planned to be transitioned to pass/fail scoring, many programs consider research productivity to be an important aspect of a resident's application. In addition, academic orthopaedic surgery departments consider research central to their mission, and contributions from residents help drive research productivity forward.

Many students have chosen to pursue research endeavors by participating in a research “gap” year. In 2009, the Association of American Medical Colleges reported that the percentage of medical students who chose to pursue nondegree research years had reached an all-time high, and the percentage of medical students who pursued a nondegree research year nearly doubled between 1999 and 2009.^2,3^ Concurrently, there has been a rise in research endeavors among residency applicants.^4^ According to the National Residency Matching Program Charting Outcomes Review, the mean number of abstracts, posters, and presentations reported by matched applicants increased over 70% between 2014 and 2018.^4^ Although the increase in both preresidency research volume and research gap years during medical school is well-documented, it is unclear whether students who participated in a research year during medical school maintain their research interest and productivity during residency. Furthermore, if a dedicated research year does not lead to continued research throughout residency, then perhaps less importance should be placed on research during the application review process. It is essential to note that although the number of publications produced may be an important factor in orthopaedic surgery residency selection, there is no evidence that the number of publications produced during residency is reflective of success during residency. This is particularly true when compared with factors such as Orthopaedic In-Training Exam scores, American Board of Orthopaedic Surgery Part I pass rate, or faculty evaluations.^5^

The purpose of this study was to answer the following questions: Do residents who participated in a research gap year during medical school publish more peer-reviewed journal publications than their peers during residency? Do residents who participated in a research gap year during medical school publish more first-author publications than their peers during residency? Which applicant characteristics are associated with a greater number of peer-reviewed journal publications produced during residency?

## Methods

### Study Design and Setting

This study was a retrospective analysis that included all orthopaedic surgery residents from two academic institutions. Residents who matriculated from June 2007 through June 2014 were included in the analysis.

### Participants/Study Subjects

All residents who matriculated during the study period were eligible for inclusion. Any residents who did not complete residency were excluded from analysis. Residents eligible for inclusion were identified by institutional databases.

### Variables, Outcome Measures, Data Sources, and Bias

Residents who completed a research gap year during medical school were identified using their Electronic Residency Application Service (ERAS) applications and confirmed by review of their curriculum vitae (CV). Resident characteristics such as USMLE step 1 score, advanced degree status, sex, and medical school were obtained from ERAS applications.

The primary outcome of interest was the number of peer-reviewed journal publications during residency. PubMed was queried to determine the number of journal publications during medical school and during residency. In an effort to account for research effect rather than solely number of publications, the journal of each publication was queried on Web of Science (Clarivate Analytics) for an impact factor number. The number of publications with a known impact factor was recorded for both cohorts.

About data sources, the CV of each resident was also reviewed to ensure accuracy. This review was done by two orthopaedic surgery residents, one medical student, and two attending orthopaedic surgeons from each involved institution.

### Description of Experiment

After Institutional Review Board exemption at the two participating institutions, the ERAS applications and CVs of the 81 residents were reviewed. In accordance with requirements from the National Residency Matching Program, each resident submitted an ERAS application during the fall of his or her fourth year of medical school with demographic information, educational background, and research listed. Each resident was also required by both residency programs to submit an updated CV in the spring of his or her last year of residency. Sex, completion of an advanced degree, participation in dedicated research gap year during medical school, medical school attended, USMLE step 1 scores, and number of podium and poster presentations before residency were collected from ERAS applications and verified with each resident's CV. Medical schools were classified into tiers based on the 2020 US News & World Report Medical School Rankings.^6^ The number of podium presentations and poster presentations during residency were collected from the CVs provided by the residents in their final year of residency.

Information regarding publications was obtained through PubMed. Similar to previous studies, each resident was searched using multiple permutations of his or her name.^7,8^ Permutations included “last name, first name”; “last name, first initial”; and “last name, first initial, and middle initial.” If multiple authors with the same name were found, publications were cross-referenced with those listed on ERAS applications and CV. Title, authorship role (first author), journal, journal impact factor, month and year of publication, and the postgraduate year of the resident (ie, Post Graduate Year 5 (PGY-5)) were recorded for each publication. Publications with only the year listed were classified as having been published in January of that year. Journal impact factor was determined using the Web of Science 2019 Journal Citation Reports (Clarivate Analytics).^9^ To account for delays between article submission and publication, articles were deemed to have been completed before residency if published in the first 6 months of internship year, and publications were deemed to have been completed during residency if published in the 6 months after residency completion. In addition, in an effort to only include work completed during residency, publications were only deemed to have been completed during residency if the work was conducted at the resident's institution or featured at least one other author from his or her institution.

### Statistical Analysis and Study Size

All statistical analyses were conducted using SPSS Statistics (version 25.0; IBM). Continuous data were presented as means and SD and categorical data as frequencies and percentages. For continuous variables, Levene's statistic determined the homogeneity of variances and the Shapiro-Wilks test of normality determined if data were normally distributed. Appropriate parametric or nonparametric tests were done. Independent samples *t*-tests or Mann-Whitney *U* tests were used to compare continuous variables between two groups. Analysis of variance or Kruskal-Wallis tests were used to compare continuous variables between three or more groups. The chi-squared test was used to test for differences between categorical variables. Finally, Pearson correlation was used to determine the relationship between the number of publications before residency and during residency. For all analyses, *P* < 0.05 was considered significant.

## Results

The study included 81 orthopaedic surgery residents who matriculated over an 8-year period (Table 1). Over 85% of the resident cohort were male. A total of 22 (27.2%) residents participated in a research gap year, and 13 (16.0%) residents had an advanced degree. A total of 49 (60.5%) residents matriculated from an institution that was defined as a top 15 medical school per US News & World Report's 2019 Medical School Research Ranking. Medical students who participated in a research year produced more publications as residents compared with their peers (22.0 ± 20.4 versus 16.5 ± 20.1; *P* = 0.022, Table 2).^10^

**Table 1:** Resident Characteristics

Characteristics	N = 81
Sex (n, %)	
Female	12 (14.8%)
Male	69 (85.2%)
Advanced degree (n, %)	13 (16.0%)
Doctor of Philosophy (PhD)	4 (4.9%)
Master of Business Administration (MBA)	3 (3.7%)
Master of Science (MSc)	3 (3.7%)
Master of Health Science (MHS)	2 (2.5%)
Master of Applied Science (MAS)	1 (1.2%)
Research year (n, %)	22 (27.2%)
Medical school tier (n, %)^a^	
1-15	49 (60.5%)
16-30	10 (12.3%)
31-45	4 (4.9%)
46-60	6 (7.4%)
>60	12 (14.8%)
Current subspecialty (n, %)	
Sports	29 (35.8%)
Hand	14 (17.3%)
Joints	12 (14.8%)
Spine	9 (11.1%)
Trauma	5 (6.2%)
Shoulder and elbow	4 (4.9%)
Foot and ankle	4 (4.9%)
Greater than one fellowship	2 (2.5%)
Pediatrics	1 (1.2%)
Tumor	1 (1.2%)

a Per 2020 US News & World Report Rankings.

**Table 2:** Factors Associated With Publications During Residency^10^

Characteristic	Mean (SD)	*P* value
Research year		**0.022**
Yes	22.0 ± 20.4	
No	16.5 ± 20.1	
Advanced degree		0.81
Yes	17.9 ± 20.2	
No	18.7 ± 21.0	
Sex		0.68
Female	17.3 ± 26.2	
Male	18.1 ± 19.2	
Medical school tier^a^		0.95
1-15	17.9 ± 18.2	
16-30	18.6 ± 27.5	
31-45	14.3 ± 6.7	
46-60	24.0 ± 30.9	
>60	16.4 ± 20.4	
Total publications before residency		**0.032**
0-5	14.8 ± 16.3	
6-10	18.3 ± 20.1	
11-15	36.5 ± 40.1	
16-20	52.0 ± 22.9	
Quartile of step 1		0.78
First	14.8 ± 16.3	
Second	18.3 ± 20.1	
Third	36.5 ± 40.1	
Fourth	52.0 ± 22.9	

a Per 2020 US News & World Report Rankings.

Bold font signifies statistical significance.

**Figure 1:**
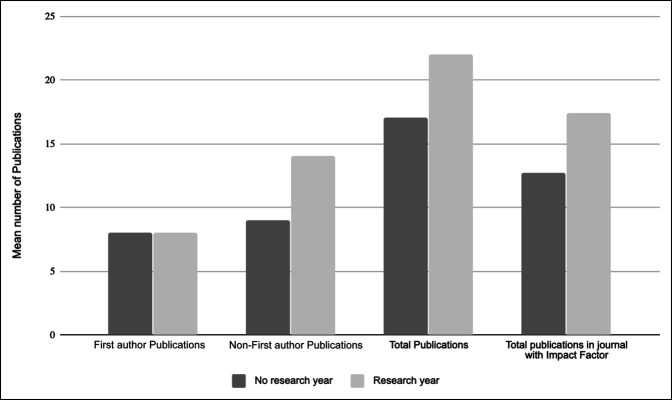
Bar graph showing the mean number of publications during residency based on participation in research year before residency.

Residents who participated in a research gap year during medical school produced more publications in journals holding an impact factor during their time in residency (12.7 ± 15.6 versus 17.4 ± 15.6, *P* = 0.022, Table 3 and Figure 1) and more non–first-author publications (8.9 ± 11 versus 14.1 ± 15, *P* = 0.027). However, the number of first-author publications was similar between those who completed a research gap year and those who did not (Table 3).

**Table 3:** Comparison of Research Output Between Residents Who Participated in a Dedicated Research Years and Their Peers Who Did Not

Factor	No Research Year	Research Year	*P* value
Publications before residency	2.9 ± 4	7.3 ± 5	**<0.001**
Publications during residency	16.5 ± 20	22.0 ± 20	**0.025**
Publications in a journal with an impact factor before residency	2.7 ± 3.4	6.8 ± 4.3	**<0.001**
Publications in a journal with an impact factor during residency	12.7 ± 15.6	17.4 ± 15.6	**0.022**
First-author publications during residency	7.6 ± 10	7.9 ± 7	0.123
Non–first-author publications during residency	8.9 ± 11	14.1 ± 15	**0.027**
Podiums before residency	1.0 ± 2.3	3.4 ± 7.3	0.516
Podiums during residency	7.6 ± 10.1	7.3 ± 9.4	0.500
Posters before residency	1.9 ± 3.4	4.0 ± 7.2	0.754
Posters during residency	6.7 ± 10.0	9.6 ± 7.1	0.306

Data reported as mean ± SD.

Bold font signifies statistical significance.

**Figure 2:**
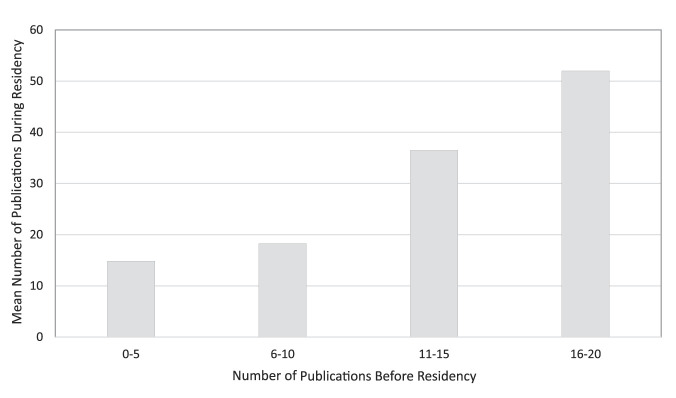
Bar graph showing the mean number of publications based on research before residency. Pearson correlation: *P* < 0.001, R = 0.363.

Other than participation in a research gap year, the number of publications before residency was correlated with the number of publications produced during residency (Pearson correlation R = 0.363, *P <* 0.001, Figure 2). Sex, medical school tier, and step 1 scores were not associated with the number of publications during residency.

**Figure 3:**
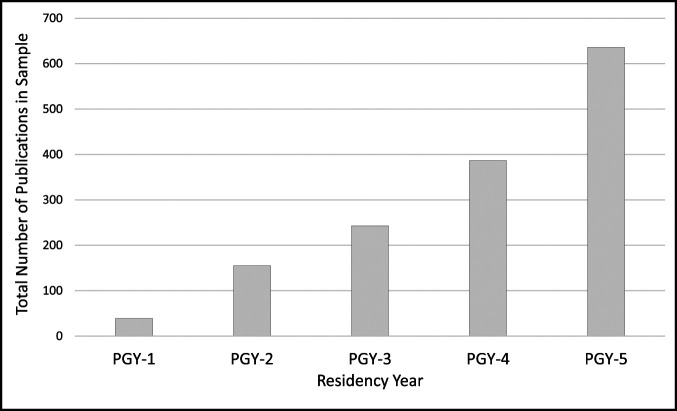
Bar graph showing the total number of publications in sample based on residency year.

No differences were found in podium or poster presentations between residents who did and did not complete a research gap year (Table 3). Before residency, the most common journals for publication were *Spine*, *American Journal of Sports Medicine*, and *Journal of Bone and Joint Surgery* (Table 4). The most common journals for publication during residency were *Arthroscopy*, *Journal of Arthroplasty*, and *Journal of Shoulder and Elbow Surgery*. The number of publications during residency increased with each subsequent year of residency (Figure 3).

**Table 4:** Most Common Journals for Publication

Before Residency			During Residency		
Journal	No. of Publications	Impact Factor (2018)	Journal	No. of Publications	Impact Factor (2018)
*Spine*	20	2.90	*Arthroscopy*	129	4.43
*Am J Sports Med*	18	6.09	*J Arthroplasty*	109	3.52
*J Bone Joint Surg Am*	18	4.72	*J Shoulder Elbow Surg*	108	2.87
*Arthroscopy*	16	4.43	*Am J Sports Med*	100	6.09
*Clin Orthop Related Res*	12	4.15	*Am J Orthop*	70	—
*J Arthroplasty*	11	3.52	*HSS J*	70	—
*J Biol Chem*	8	4.11	*J Bone Joint Surg Am*	69	4.72
*Orthopaedics*	7	1.61	*Orthop J Sports Med*	57	
*J Orthop Trauma*	7	1.83	*Orthopaedics*	53	1.61
*Knee Surg Sports Traumatol Arthrosc*	7	3.15	*Spine*	53	2.90
*Am J Orthop*	6	—	*J Hand Surg Am*	42	2.09
*J Pediatr Orthop B*	6	0.74	*Arthrosc Tech*	41	—
*Knee*	5	1.76	*Clin Orthop Related Res*	41	4.15
*J Biomed Mater Res A*	5	3.22	*Foot Ankle int*	36	2.34
*J Neurosci*	5	6.07	*J Orthop Trauma*	32	1.83
*J Shoulder Elbow Surg*	4	2.87	*JBJS Case Connect*	28	—
*J Hand Surg Am*	4	2.09	*J Am Acad Orthop Surg*	26	2.35
*J Knee Surg*	4	1.59	*Sports Health*	18	2.65
*Biomaterials*	4	10.27	*Arch Orthop Trauma Surg*	17	1.97

After multivariate regression analysis, when controlling for USMLE step 1 score, US News & World Report Medical School ranking, and sex, students who participated in a research gap year were 11 times more likely to be in the top 50th percentile in the number of peer-reviewed publications produced during residency (Table 5). After multivariate regression, USMLE step 1 scores, Medical school tier, or sex were not independently associated with an increased number of publications during residency.

**Table 5:** Factors Associated With Publications During Residency With Multivariable Regression

Characteristic	Odds Ratio	95% Confidence Interval	*P* value
Research year	11.1	2.3-53.7	0.003
Step 1 score	1.03	0.98-1.08	0.207
Medical school tier			
1 (reference)			0.51
2	0.88	0.20-3.8	0.86
3	0.22	0.02-2.4	0.99
4	0.98	0.09-10.8	0.21
5	2.79	0.29-27.3	0.99
Male sex	2.57	0.54-12.28	0.24

## Discussion

Matching into an orthopaedic surgery residency program has become increasingly difficult, with the applicants possessing among the highest USMLE step 1 scores, clinical grades, and number of research publications of the various residency specialties.^1,2^ This has led to an increase in the number of medical students participating in nondegree research years; however, the value of these research years and their effect on research productivity during residency remained unknown.^2,11^ Our study found that residents who had completed a research year during medical school produced more peer-reviewed journal publications during residency than their peers who had not. However, this may be due to a variety of factors, including greater familiarity and experience with research gained during the year, self-selection bias with residents who were more interested in research electing to partake in a research year, and/or established relationships with research mentors if they conducted their research year at their ultimate residency institution. It is also crucial to note that the number of peer-reviewed publications produced by a resident is not reflective of the quality of education of a residency program or the clinical development of the individual resident. Contextualizing the association of research productivity on success in residency is important because factors such as Orthopaedic In-Training Exam scores, American Board of Orthopaedic Surgery Part I pass rate, and faculty evaluations all play a role in determining success in residency. In fact, future studies may investigate whether research productivity affects overall resident education. Nonetheless, research involvement during residency is important because it may advance the field of orthopaedics, improve one's understanding of research methodology and, in turn, comprehension of research articles, and may enable some residents to pursue academic careers.^12^ As expected, residents who conducted a research year had a markedly greater number of overall publications. However, we found no difference in the number of first-author publications among the groups. This may suggest that although research-year residents may engage in more projects that culminate in publications than their peers, the nature of their roles in these projects may be similar.

This study has a number of limitations. The primary outcome of interest was the number of journal publications found on PubMed. This analysis did not include peer-reviewed publications that are not indexed in PubMed, book chapters, or non–peer-reviewed publications, all of which reflect to academic productivity. The number of journal publications was used as a proxy for research productivity, and although we included impact factor to emphasize that quality is as important as quantity of research, the number of publications does not necessarily reflect the effect of a resident's research. Importantly, not all publications represent equivalent levels of research because distinctions exist between case reports, review articles, and scientific research with a stated purpose and hypothesis. This study featured data from two academic institutions, and the number of peer-reviewed publications may not be reflective of data captured from nonacademic institutions. In addition, there is often a delay from completion of a project to publication date, and some research included as having been completed during residency may be research from medical school. Finally, the accuracy of CVs, ERAS applications, poster, and podium data is dependent on the validity of residents' self-reports.

Literature regarding predictors of research productivity during residency remains equivocal. In their analysis of 147 residents at a single institution, Spitzer et al^13^ found that the number of articles published before residency was positively associated with the number of articles published during residency. Kreitz et al^14^ surveyed 13 orthopaedic residency programs and found that preresidency first-author publications, honors in >5 clerkships, and an undergraduate basic science major markedly increased the odds of publishing as a first author during residency. Interestingly, they found that USMLE step 1 and step 2 scores were negative predictors—meaning that residents with higher test scores were less likely to publish a first-author publication than their lower scoring peers.^14^ Notably, however, there was no evaluation of whether the residents in these two studies participated in a formal research year. Although our findings, along with those of Spitzer et al. and Kreitz et al., found that the number of overall publications before residency was markedly associated with the number of publications during residency, this relationship remains unclear in other subspecialties. A study evaluating otolaryngology residents found that residents who had at least one publication before residency were six times more likely to publish during residency than those that had not, whereas other studies evaluating radiology, urology, and radiation oncology residents did not find a notable relationship.^8,15-17^

Although research years may increase the number of publications a resident publishes during his or her residency, the benefit of these research years for medical students and their effect on careers remain unknown. In a 2020 study that surveyed 34 research year participants, 85.3% of respondents reported becoming more competitive for top-tier orthopaedic residencies as one of their reasons for participating in a research year.^2^ This suggests that medical students who participated in a research gap year do so for reasons other than necessarily wanting to pursue a career in academics or even an interest in research. Sidiqi et al^18^ noted in a 2016 survey of applicants for Radiology-Oncology, another competitive subspecialty, 68% of students stated that the primary purpose of their research gap year was to obtain more publications. The suggestion that students who participated in a research year do so to improve their application parallels the findings of Egol et al.^11^ who found that gap-year students have slightly lower USMLE step 1 scores (236 versus 240) and USMLE step 2 scores (243 versus 247). The belief in the importance of a research year, however, did not entirely align with those of the 72 program directors surveyed with only 3 (4.2%) agreeing that a research year was an important factor for residency selection. In a recent study that surveyed 78 program directors, research was ranked as the 11th most important factor in evaluating applicants, even below that of personal appearance which ranked 10th.^19^ However, a single-center, retrospective study found that 57 of 58 medical students who conducted a research year at their academic institution during medical school successfully matched into orthopaedic surgery—a rate that was significantly higher than the national average during this period (98.3 versus 67.9, *P* < 0.001). Similarly, it remains unclear whether research years and their associated increased research productivity lead to more residents entering academic positions and continuing to conduct research. In a study that featured residents who participated in a dedicated research year *during* residency, Segal et al.^20^ found that although residents who participated in a dedicated *residency* research year had markedly more publications than their peers, they were not more likely to enter academics after this experience. Similarly, Torres et al.^21^ found that although a dedicated research program during residency increased the quantity and mean impact factor of research, this productivity did not persist beyond residency. Future research should evaluate whether residents who participated in a research gap year during *medical school* are more likely to continue their research after residency and may help elucidate research should be considered as an important factor in determining residency selection, especially at academic institutions.

## Conclusion

Selecting orthopaedic surgery residents has become an increasingly challenging task with a growing number of applicants electing to participate in research years to bolster their applications. Our study found that research years were associated with a greater number of overall publications and more publications in journals with impact factors during residency. However, noteworthy is that this does not suggest in any way that increased research productivity translates to increased residency education. However, additional work is required to evaluate whether this increased productivity persists after residency.
